# Physical deterioration and adaptive recovery in physically inactive breast cancer patients during adjuvant chemotherapy: a randomised controlled trial

**DOI:** 10.1038/s41598-020-66513-9

**Published:** 2020-06-16

**Authors:** Tom Møller, Christina Andersen, Christian Lillelund, Kira Bloomquist, Karl Bang Christensen, Bent Ejlertsen, Malgorzata Tuxen, Peter Oturai, Ulla Breitenstein, Cecilie Kolind, Pernille Travis, Tina Bjerg, Mikael Rørth, Lis Adamsen

**Affiliations:** 1 The University Hospitals Centre for Health Research (UCSF), Copenhagen University Hospital, Rigshospitalet, Department, 9701 Copenhagen, Denmark; 2 Copenhagen University Hospital, Rigshospitalet, Department of Oncology, 7301 Copenhagen, Denmark; 3 Copenhagen University Hospital, Herlev Hospital, Department of Oncology, Herlev, Denmark; 4 University of Copenhagen, Faculty of Health and Medical Sciences, Department of Public Health, Copenhagen, Denmark; 5 Copenhagen University Hospital, Rigshospitalet, Department of Clinical Physiology, Nuclear Medicine and PET, Copenhagen, Denmark

**Keywords:** Quality of life, Translational research, Breast cancer

## Abstract

Cardiorespiratory fitness is an independent risk factor for cardiovascular disease and shortened life expectancy in breast cancer survivors. This randomised controlled trial (n = 153) was designed for patients with a physically inactive lifestyle prediagnosis and concurrently referred to adjuvant chemotherapy. We compared two 12-week exercise interventions aimed at physiological and patient-reported outcomes (cardiorespiratory fitness, muscle strength, metabolic markers, physical activity, pain, fatigue), including a 39-week follow-up. A supervised hospital-based moderate to high intensity group exercise intervention was compared to an instructed home-based individual pedometer intervention. The two 12-week interventions included oncologists’ recommendations and systematic health counselling. Outcomes were measured at baseline and week 6, 12 and 39. Primary outcome cardiorespiratory fitness declined significantly during chemotherapy and was restored in both interventions at follow-up. The interventions effectively engaged breast cancer patients in sustaining physical activities during and following adjuvant treatment. A composite metabolic score improved significantly. Positive cardiorespiratory fitness responders had improved clinical effects on fatigue, pain and dyspnoea versus negative responders. We conclude that a loss of cardiorespiratory fitness among physically inactive breast cancer patients may be restored by early initiated interventions and by adapting to physical activity recommendations, leading to a decreased cardiovascular risk profile in breast cancer survivors.

## Introduction

Physical inactivity and sedentary behaviour represent a challenge for global health due to a significant risk of pre-mature death from chronic diseases such as cardiovascular diseases, diabetes and cancer^1,2^. In breast cancer (BC), physical inactivity and obesity has consistently been associated with an increased risk of recurrence^3,4^, though physiological, biological and molecular pathways are partly known^5–8^. Low cardiorespiratory fitness (CRF) increases risk for coronary heart disease and affects survival in BC survivors^9^, similar to asymptomatic women^10^, low-risk adults^11^ and high-risk sedentary populations^12,13^. Compared with healthy sedentary Americans, a relative 25% decline in CRF has been shown to be a primary recurring effect in the BC trajectory^14^, a finding confirmed in a German cross-sectional study^15^. The causal mechanisms involved in this decline in CRF in patients with BC may be related to a cascade of factors in the oxygen delivery system^15–17^. These factors may include direct chemotherapy toxic impairment on the left ventricular function and endothelial damage, in combination lifestyle factors with a negative effect, such as physical inactivity and weight gain^17,18^.

Various studies point out the importance of having built-in, regular physical activity (PA) in multimodal cancer supportive care and rehabilitation^19–21^. There appears to be a potential health gain related to moderate and high intensity PA, with the highest effect on CRF and mortality observed in individuals performing high intensity exercise compared to controls or sedentary populations^22,23^. A recent systematic review on cancer survivors^24^, e.g. sedentary BC survivors^25^, found that walking was the strongest PA modality preference. Nonetheless, less attention has been paid to recruiting physically inactive cancer patients in exercise programmes^26^. It remains unclear what the optimal setting, timing during cancer trajectory, dosage and combination of exercise and health-promoting components that best facilitate patient adherence and symptom management to support physiological improvements and sustainable lifestyle changes in a physically inactive BC population^27^. Prevention of a decline in CRF may have a salutogenic medical effect that necessitates the integration of lifestyle modifications in BC oncology^28–30^ that specifically support behavioural aspects when recommended PA guidelines are not met.

However, recent randomised controlled trials (RCTs) among patients with BC have raised concerns about the potential interventional effect on CRF during adjuvant chemotherapy and at follow-up^23,31,32^. This concern appears to derive from a shift in chemotherapy regimens incorporating taxane-based chemotherapy, implicating an altered patient symptom profile^33,34^. In the randomised feasibility study preceding the present RCT, we used Danish national PA guidelines as the initial screening inclusion criteria^35^. This simplified two-item tool was able to detect physically inactive BC patients at onset of adjuvant chemotherapy with a high correlation of low VO_2_peak at baseline compared with the Scandinavian background population^27^. Qualitative in-depth studies indicate that a recommendation from clinicians to exercise at the specific timepoint around chemotherapy onset is ideal for recruiting subjects and initiating PA^36^. Moreover, we proposed an overall framework for transforming behavioural change towards re-thinking PA into post-cancer life priorities in an at-risk, physically inactive BC population^36^.

The present RCT aimed to compare the effects of two 12-week exercise interventions on physiological outcomes (e.g. CRF, muscle strength, body composition, blood cholesterol and insulin) and patient-reported outcomes (e.g. PA, pain, fatigue, dyspnoea and anxiety), including a 39-week follow-up. The population comprised screened, physically inactive BC patients at onset of adjuvant chemotherapy that included sequential anthracycline and cyclophosphamide with docetaxel or paclitaxel-based regimens.

## Patients and methods

### Research design and study population

This study was designed as an assessor single-blinded two-centre RCT comparing a structured 12-week supervised hospital-based group exercise intervention (Group 1 (Gr. 1)) versus a 12-week instructed home-based individual pedometer intervention (Group 2 (Gr. 2)) during adjuvant chemotherapy among confirmed physically inactive patients with BC. Both interventions were combined with health and symptom guidance given by a clinical nurse specialist. The primary outcome of interest was CRF (CRF/VO_2_peak). The RCT was analysed and published according to Consolidated Standards of Reporting Trials (CONSORT) guidelines^37^.

Patients who had symptomatic heart disease, bone metastasis, received neo-adjuvant chemotherapy, suffered from psychotic conditions or did not understand Danish were excluded. Patients who met the national criteria for leisure time PA were not eligible for the study^27,35,38^.

### Setting

This study was conducted at the Departments of Oncology, Copenhagen University Hospital, Rigshospitalet and Herlev Hospital at the Centre for Integrated Rehabilitation of Cancer Patients (CIRE), Copenhagen, Denmark and was established and supported by the Danish Cancer Society and the Novo Nordic Foundation. CIRE adheres to three key intervention principles: (1) **e**arly initiation of an intervention during cancer treatment; (2) **ex**ercise/physical activity and (3) patient **act**ivation (EEX-ACT)^39^.

### Approvals

#### Ethics approval and consent to participate

All patients provided informed written consent and all methods were performed in accordance with the relevant guidelines and regulations. This study was approved by the Scientific Committee of the Capital Region of Denmark (file no. H3-2013-155), and the Danish Data Protection Agency (file no. 2011-41-6349). Trial registration: Current Controlled Trials 13/03/2014 ISRCTN13816000.

### Procedure

**Figure 1:**
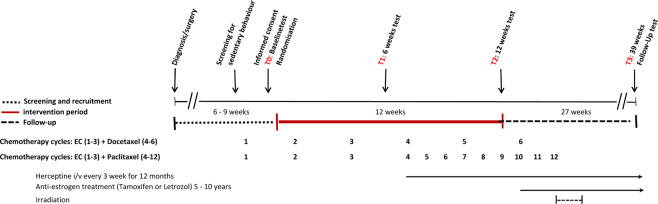
Global study overview during chemotherapy.

Eligible BC patients were identified with a pre-screening instrument based on PA guidelines from the Danish Health and Medicines Authority (>150 minutes of regular and moderate recreational PA and at least 2 × 20 min of strenuous exercise per week)^35^. The oncologists or primary nurse present during the patients’ adjuvant chemotherapy provided the initial screening assessment of these two PA parameters. A clinical nurse specialist on the exercise team subsequently gave in-depth information to physically inactive BC patients about the study’s rationale, tests and intervention. Baseline testing was planned between the first and second cycle of epirubicin and cyclophosphamide (see Fig. 1). Patients were stratified by age (<48>) and hospital setting and then randomly assigned 1:1, either to Gr. 1 or Gr. 2, by the Copenhagen Trial Unit. Informed consent was obtained from every participant included in the study.

### Interventions

The protocol for this study described and compared the two pragmatic interventions in more detail^38^, while the feasibility study investigated the validity of the PA screening tool related to the primary outcome (CRF), patient acceptance, interventional safety, adherence and programme feasibility^27,36^.

#### Supervised exercise intervention

12-week supervised hospital-based group exercise intervention and health counselling and symptom guidance.

Patients were offered a 12-week supervised exercise programme (PART1: six weeks, 9 h/week and PART2: six weeks, 6 h/week) in groups of 10–14 patients supervised by an exercise physiologist/physical therapist and a clinical nurse specialist. PART1 included three training sessions per week and one restorative session comprising high-low-intensity components (cardiorespiratory training on stationary bikes, resistance training, relaxation training and massage). PART2 comprised the sports floorball games, dance and circuit training. The total training volume in PART1 and PART2 corresponded to approximately a 40-metabolic equivalent of task hours per week^19^. Pre-exercise safety screening took place before each session and involved moderate-to-high-intensity physical training components^19,38^.

#### Instructed pedometer intervention

Individual 12-week instructed home-based pedometer intervention and health counselling and symptom guidance.

The instructed pedometer intervention was a 12-week individually organised programme designed to progressively support increased PA and was predominantly provided by a clinical nurse specialist in cancer and exercise. Patients were encouraged to enhance their PA levels and to avoid physical inactivity by integrating exercise into activities of daily living. The initial explicit goal was to achieve a low to moderate recreational PA level of 30 min/day (aerobic walking) and, stepwise, to achieve 7,500 steps/day five times per week, with the ultimate goal of incorporating 150 minutes of moderate-to-vigorous PA per week^40,41^. The Omron Walking Style Pro 2.0 Pedometer made it possible to visually portray the patients’ exercise achievements on a daily, weekly and monthly basis at a scheduled instruction and evaluation meeting baseline at weeks 2, 4, 6, 9 and 12. Pedometer data were transferred electronically to investigators.

#### Health counselling and symptom guidance

Patients in both interventions received individual test feedback and health counselling and symptom guidance at baseline and at weeks 6, 12 and 39 to help counteract barriers and to support individual motivational aspects to initiate and prioritise leisure time PA^27,38^. The theoretical framework for initiating and adopting behavioural change at onset and during adjuvant chemotherapy focused on recognising pre-exercise history, individual goal planning, family resources and the immediate pitfalls during anticancer treatment as crucial determinants for the individual to rethink regular exercise as pivotal for their health and post-cancer life priorities^36^.

### Assessments, outcomes and data protection

Patient assessment of primary outcome, CRF/VO_2_peak, and secondary outcomes was conducted at time of inclusion, baseline (**TO**); week 6, midway (**T1**); week 12, completed (**T2**); and week 39, follow-up (**T3**) (six months after intervention and chemotherapy and irradiation completed) (Table 1). Assessments were blinded to test personnel (affiliated health professionals trained by an exercise physiologist). Fasting, whole-body dual-energy X-ray absorptiometry was performed by medical technicians who were not involved in group allocation or interventional activities at Copenhagen University Hospital, Rigshospitalet, Department of Clinical Physiology, Nuclear Medicine and PET. Figure 1 provides a global study overview.

**Table 1:** Outcome assessments, instruments and timepoints.

ASSESSMENT	INSTRUMENT/METHOD	TIMEPOINT
Primary outcome Cardiorespiratory fitness/oxygen uptake	Maximal oxygen uptake (VO_2_peak); incremental test on cycle ergometer (Monark Ergomedic 839E) with direct measures of respiratory gases^27,38^ Assessment of physical exertion on physiological tests (1.10 and >1.15) and the Borg Scale^42^	T0, T1, T2, T3 T0, T1, T2, T3
**Secondary physiological outcomes**		
Body composition (fat mass, lean mass, bone density)	Fasting whole-body DXA scan (fat percent, visceral fat, lean mass, android/gynoid ratio, bone mass density^43,44^	T0, T2, T3
Muscle strength	Isokinetic maximum knee extension strength (60^0^/sec.) (Contrex MJ Isokinetic Dynamometer CMV AG, Switzerland) Maximum load (one repetition maximum = 1RM) measured at knee extension, leg press and lateral pull (Technogym (Gambettola, Italy)	T0, T1, T2, T3 T0, T1, T2, T3
Biomarkers	Lipids, cholesterols, blood glucose, insulin^14^	T0, T2, T3
Metabolic syndrome profile	Composite score: cholesterol, triglyceride, blood glucose, blood pressure, android/gynoid (AG) ratio^45^	
Blood pressure	Test, A&D Medical UA-852 Digital Blood Pressure Monitor	T0, T1, T2, T3
Haemoglobin	Test	T0, T1, T2, T3
Physical activity (objective)	Pedometer in home-based exercise programme, Omron Walking Style Pro 2.0^27^	T0 to T2 continuous (solely pedometer group)
Pulse	Pulse sensor during supervised exercise intervention, Polar Team System 2, Polar, Finland	T0 to T2 continuous (supervised exercise group only)
**Psychometric measures**		
Health-related quality of life	European Organization for Research and Treatment of Cancer Quality of Life Questionnaire^46^	T0, T1, T2, T3
Anxiety and depression	Hospital Anxiety and Depression Scale^47^	T0, T1, T2, T3
Physical activity (subjective), labour market, lifestyle factors (smoking, alcohol, diet)	Self-developed questionnaire with guideline-based physical activity scale^35^	T0, T1, T2, T3
Clinical characteristics and treatment	Medical records	Continuous
Diagnosis, stage, anti-cancer treatment		

Abbreviations: TO = baseline; T1 = week 6, midway; T2 = week 12, completed; T3 = week 39, follow-up; DXA = dual-energy X-ray absorptiometry; 1RM = one repetition maximum.

All outcomes were entered into a secure database with tracking hosted by the Copenhagen Trial Unit at Copenhagen University Hospital. Data access for study researchers was provided when the last sequentially numbered patient had completed the T2 assessment (Table 1).

### Power calculation and statistics

Based on the a priori assumption that the supervised exercise intervention will be more effective in maintaining or increasing aerobic capacity (VO_2_peak), sample size was based on an expected mean difference between groups of VO_2_peak 180 ml/min and with a standard deviation (SD) of 24^19,23,27^. With a power of 0.9, a dropout rate of 25% and assuming that the population would comprise 70% true non-training, physically inactive patients (VO_2_peak measure compared against the background population)^48^, the final sample included n = 77 in each study arm, yielding 154 patients.

The principal analyses of primary and secondary outcomes employed the intention-to-treat approach by including all available data. Data were reported as means and SDs. Change scores and differences between change scores were derived from the linear mixed model along with corresponding 95% confidence intervals using the delta method. No attempts at imputation beyond those implicit in linear mixed models were implemented for subjects with missing follow-up data, but pattern mixture models were performed for the primary outcome dropout analyses^49^. Baseline demographic characteristics were provided with 95% confidence intervals.

## Results

### Patients

**Figure 2:**
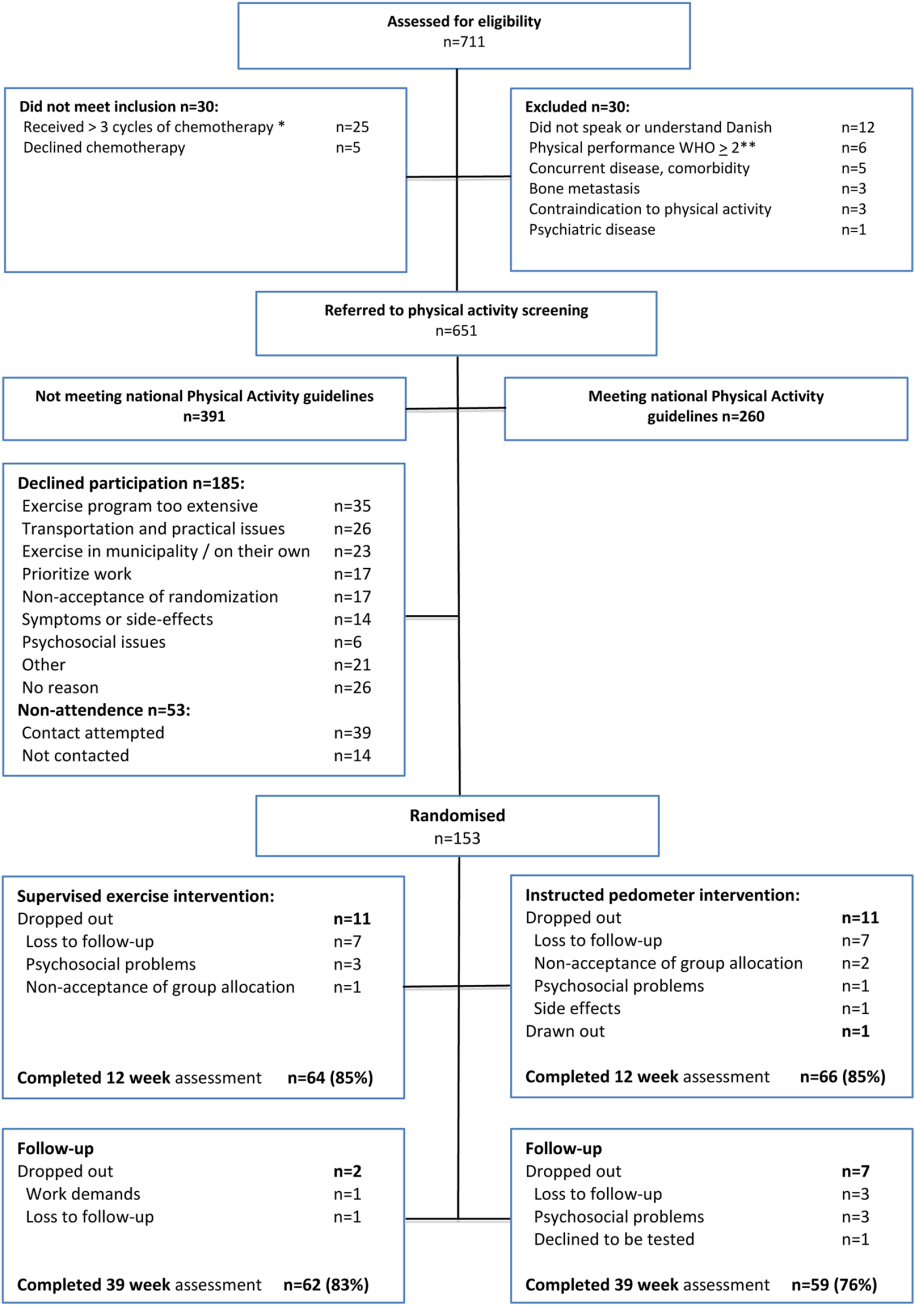
Consort flow chart: recruitment and completion.

A total of 711 patients were screened for eligibility, 60% of whom were considered physically inactive prior to their diagnosis, while 40% met national PA recommendations. Study recruiters failed to establish contact with 53 patients (13.5%). With 185 patients declining to participate, the study acceptance rate was 45%. The study attrition rate was 15% for each study group at T2. At T3 it was 17% for Gr. 1 and 24% for Gr. 2 (Fig. 2). Test adherence at week 12 was 85% in both the supervised exercise and the instructed pedometer interventions. Programme adherence measured for 12-week completers was 1617/2304 or 70.2% for supervised exercise participation and 378/396 or 95.5% for PA pedometer instruction meetings.

No adverse events prompting medical attention occurred during the supervised exercise sessions. Three minor training-related injuries occurred (pulled muscle, sprained foot, back problem). Two patients experienced a lower dyspnoea threshold during moderate intensity training with no further action taken. Another two minor adverse events (temporary swelling and foot pain) related to the instructed pedometer intervention were reported.

The stratified randomisation procedure balanced baseline characteristics across groups (Table 2). Five patients (3%) reported doing some moderate to strenuous PA, while approximately one-third (36%) reported doing low to moderately active PA > 150 minutes per week. At baseline, 90% had a VO_2_peak lower than the age-matched background population of Scandinavian women. Participants received four courses of chemotherapy during the intervention period consisting of one or two courses of epirubicin and cyclophosphamide followed by two or three courses of docetaxel-based chemotherapy. For nineteen participants, one series of docetaxel was replaced with three series of paclitaxel as standard treatment, equally distributed between groups.

**Table 2:** Baseline characteristics.

	Total (N = 153)	Supervised hospital- based exercise intervention (n=75)	Instructed pedometer intervention (n=78)	diff(95% confidence interval)
Age, mean ± SD (y)	51.7 ± 9.4	51.5 ± 9.6	52.0 ± 9.3	−0.5 (−3.5 to 2.5)
BMI, mean ± SD (kg/m2)	26.1 ± 5.1	26.2 ± 5.3	26.0 ± 4.9	0.2 (−1.4 to 1.9)
**Marital status n (%)**				
Single/divorced/widowed	50 (33)	26 (35)	24 (31)	3 (−12 to 18.1)
Married/living together	103 (67)	49 (65)	54 (69)	−4 (−18.1 to 12)
**Education n (%)**				
Lower	12 (7.9)	7 (9.3)	6 (7.7)	1.6 (−8.1 to 9.2)
Secondary	48 (31.8)	25 (33.3)	24 (30.8)	2.5 (−12.8 to 17)
Advanced	91 (60.3)	43 (58.9)	48 (61.5)	−2.6 (−18.3 to 13)
**Smoking n (%)**				
Never/ex-smoker*	138 (90.8)	68 (90.7)	70 (89.7)	1.0 (−9.4 to 9)
Current	15 (9.8)	7 (9.3)	8 (10.3)	−1.0 (−9 to 9.4)
**Alcohol**				
Intake per week, median	2 (0 to 5.5)	2 (1 to 6.5)	2 (0 to 5)	0 (0 to 1)
**Physical activity. Prediagnosis No. (%)**				
Light-moderate PA <150 min/week	99 (65)	44 (59)	55 (71)	−12 (−27 to 3.2)
Light-moderate PA >150 min/week	54 (35)	31 (41)	23 (29)	11.8 (−3.2 to 27)
Strenuous activity <2 × 20 min/week	148 (97)	73 (97)	75 (96)	1.2 (−4.4 to 6.8)
Strenuous activity >2 × 20 min/week	5 (3)	2 (3)	3 (4)	−1.2 (−6.8 to 4.4)
**Cancer stage, n (%)**				
Stage 1	56 (36.6)	31 (41.3)	25 (31.1)	9.3 (−5.9 to 24.5)
Stage 2	81 (52.9)	36 (48.0)	45 (57.7)	−9.7 (−25.4 to 6.1)
Stage 3	16 (10.5)	8 (10.7)	8 (10.3)	0.4 (−9.3 to 10.1)
**Breast surgery, n (%)**				
Lumpectomy	90 (58.8)	47 (62.7)	43 (55.1)	7.5 (−8 to 23.1)
Mastectomy	56 (36.6)	26 (34.7)	30 (38.5)	−3.8 (−19 to 11.5)
Mastectomy plus expander	7 (4.6)	2 (2.7)	5 (6.4)	−3.7 (−10.3 to 2.8)
**Chemotherapy, n (%)**				
Q3Wy CE × 3 -> Q3W docetaxel × 3	130 (85.0)	66 (88.0)	64 (82.1)	5.9 (−5.3 to 17.2)
Q3W CE × 3 -> weekly paclitaxel × 9	19 (12.4)	8 (10.7)	11 (14.1)	−3.4 (−13.9 to 7)
Other	4 (2.6)	1 (1.3)	3 (3.9)	−2.5 (−7.5 to 2.5)
Pegfilgrastim n (%)	114 (75)	56 (75)	58 (74)	
Radiotherapy, n (%)	121 (79.1)	58 (77.3)	63 (80.8)	−3.4 (−16.3 to 9.5)
Herceptin, n (%)	44 (28.8)	22 (29.3)	22 (28.2)	1.1 (−13.2 to 15.5)
Endocrine treatment, n (%)	118 (77.1)	59 (78.7)	59 (75.6)	3.0 (−10.3 to 16.3)
Tamoxifen	62 (41)	33 (44)	29 (37)	—
Letrozole	44 (29)	21 (28)	23 (29)	—
Other	12 (8)	5 (7)	7 (9)	—

Abbreviations: diff=difference; BMI = body mass index; *cessation>1 year; Q3W = every three weeks; CE = cyclophosphamide and epirubicin.

### Primary outcome

**Figure 3:**
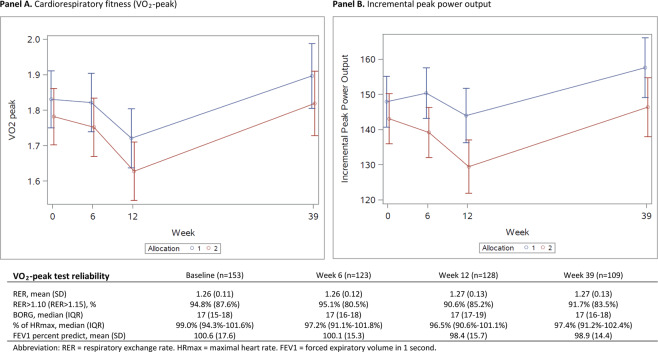
Primary outcome.

The incremental test on the cycle ergometer with direct measures of respiratory gases was performed with a high reliability (Fig. 3). The primary outcome, VO_2_peak, decreased significantly in both study groups (Gr. 1: 6.2%, Gr. 2: 9.1%) from baseline to week 12 (T2) without any between-group difference. Both study groups restored VO_2_peak from week 12 to week 39 (p < 0.0001) with no significant between-group differences observed (Fig. 3, Panel A). There was a higher attrition rate in Gr. 2 among 12 patients with lower mean VO_2_peak baseline values, which is in accordance with the power calculation and exceeded the minimal clinical relevance of 180 ml/min at week 12. However, a dropout analysis at week 12 showed no differences in socio-demographics or treatment characteristics among completers and non-completers. At week 39 the difference in baseline VO^2^peak between completers and non-completers did not exceed the minimal clinically important threshold.

The incremental peak power output (IPPO) differed significantly between study groups, favouring the supervised exercise intervention but did not remain significant at T3 (Fig. 3, Panel B). Similar to VO_2_peak measures, the mean baseline IPPO was lower among patients with no follow-up assessments.

### Secondary outcomes

#### Self-reported physical activity level

**Figure 4:**
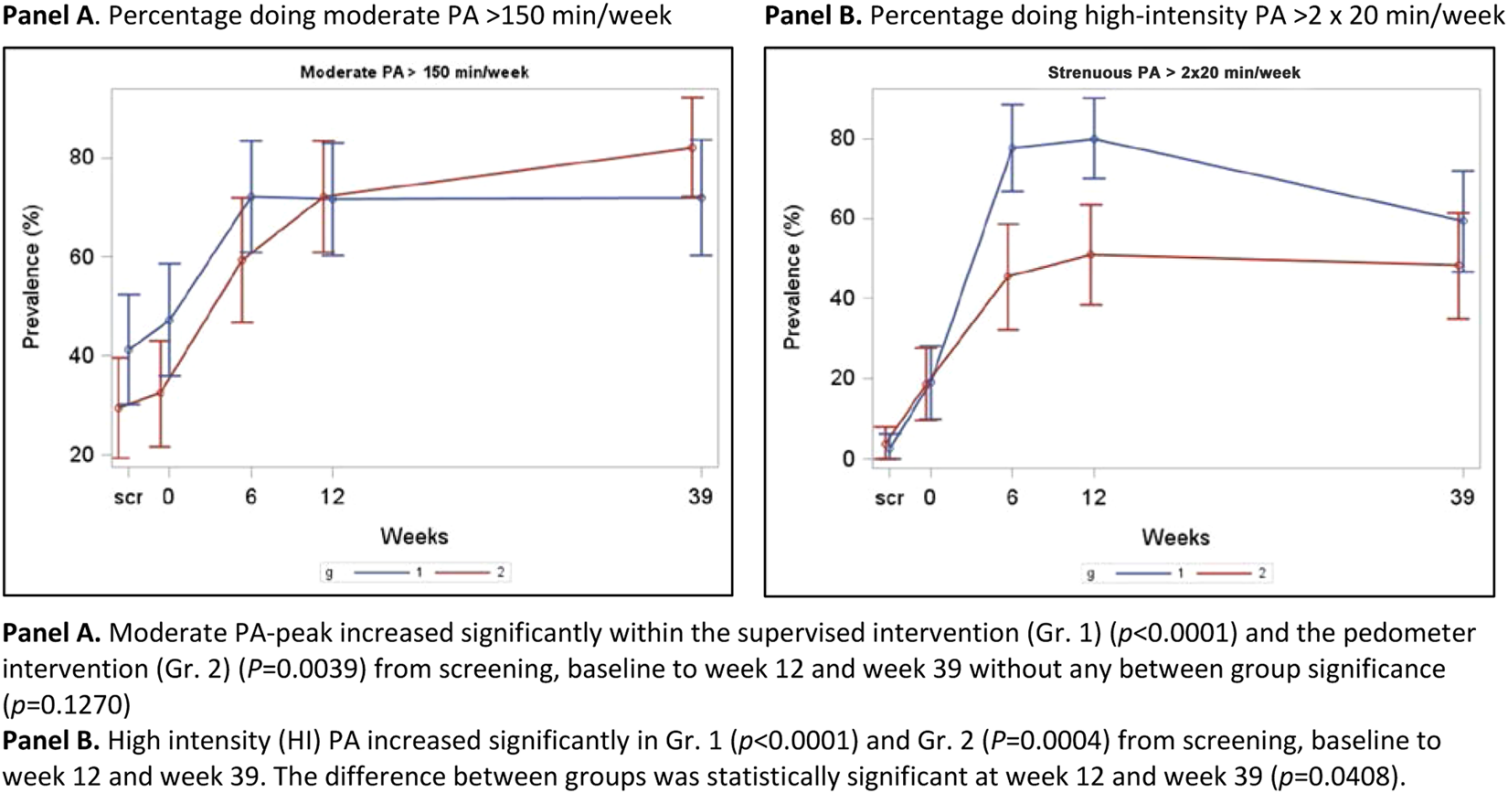
Percentage doing moderate and high intensity physical activity.

Figure 4 Panels A and B presents the self-reported prevalence in PA in accordance with Danish national PA guidelines. Both study groups showed a highly significant increase in moderate PA from baseline (T0) to intervention completion (T2) that remained at the week-39 follow-up (T3). The incorporation of high-intensity PA components was increased significantly in both study groups; however, there was a significant difference in favour of the supervised exercise intervention compared to the instructed pedometer intervention during the 12-week period and at T3 (*p* = 0.0408). At T3, more than 50% met the national PA guidelines for adults.

#### Muscle strength and isokinetic measures

The supervised exercise intervention showed within-group increases in strength using the one repetition maximum test (knee extension, lateral pull and leg press), with significant between-group differences visible at intervention completion (T2) and sustained at follow-up (T3). The isokinetic measures significantly favoured the supervised exercise intervention when it concluded (T2), but there was no between-group significance at week 39 (T3), where baseline isokinetic measures were restored (Table 3).

**Table 3:** Strength (1RM) and isokinetic measures (Contrex).

Variable	Allocation	T0 (n = 153)	T1 (n = 124)	T2 (n = 127)	T3 (n = 112)	(95% CI)	p	Between group	p
	group	Mean (SD)	Mean (SD)	Mean (SD)	Mean (SD)			diff kg (95% CI)	
1RM knee extension (kg)	1	39 (10)	47 (10)			8 (6 to 10)	<0.0001	8 (5 to 10)	*<0.0001
	2	41 (12)	41 (12)			0 (−2 to 2)	0.8138		—
	1	39 (10)		49 (10)		10 (7 to 12)	<0.0001	8 (4 to 11)	*<0.0001
	2	42 (13)		43 (13)		2 (−1 to 4)	0.1493		—
	1	39 (9)			46 (10)	7 (4 to 9)	<0.0001	6 (2 to 9)	*0.0022
	2	41 (12)			43 (12)	1 (−1 to 4)	0.3197		—
1RM lateral pull (kg)	1	29 (7)	33 (7)			4 (2 to 6)	<0.0001	3 (1 to 5)	*0.0173
	2	30 (8)	32 (7)			1 (−1 to 3)	0.168		—
	1	30 (7)		36 (7)		6 (4 to 8)	<0.0001	4 (1 to 7)	*0.0050
	2	30 (8)		32 (7)		2 (0 to 4)	0.0525		—
	1	29 (7)			33 (6)	4 (2 to 6)	0.0001	3 (0 to 6)	*0.0287
	2	30 (7)			31 (7)	1 (−1 to 3)	0.4116		—
1RM leg press (kg)	1	84 (27)	106 (33)			21 (16 to 26)	<0.0001	20 (13 to 27)	*<0.0001
	2	85 (36)	86 (38)			1 (−4 to 6)	0.6769		—
	1	84 (27)		108 (36)		24 (18 to 29)	<0.0001	22 (14 to 29)	*<0.0001
	2	86 (36)		87 (38)		2 (−3 to 7)	0.4468		—
	1	82 (26)			103 (37)	21 (16 to 27)	<0.0001	18 (10 to 26)	*<0.0001
	2	84 (35)			87 (36)	3 (−3 to 8)	0.2951		—
Contrex Peak right	1	117 (23)	118 (23)			1 (−2 to 4)	0.4679	4 (0 to 8)	0.0518
	2	119 (29)	116 (29)			−3 (−6 to 0)	0.0447		
	1	118 (23)		118 (22)		1 (−3 to 4)	0.6659	6 (1 to 11)	*0.0126
	2	120 (29)		114 (27)		−5 (−9 to −2)	0.0019		
	1	116 (23)			115 (23)	−1 (−5 to 3)	0.5158	2 (−3 to 8)	0.422
	2	120 (30)			116 (29)	−4 (−8 to 0)	0.0752		
Contrex Peak left	1	116 (23)	120 (21)			4 (1 to 6)	0.0191	5 (1 to 9)	*0.0246
	2	119 (29)	117 (28)			−1 (−4 to 2)	0.3916		
	1	116 (24)		118 (24)		2 (−2 to 5)	0.2956	6 (1 to 11)	*0.0269
	2	119 (30)		115 (30)		−4 (−7 to 0)	0.0354		—
	1	116 (22)			116 (22)	1 (−4 to 5)	0.7467	4 (−2 to 11)	0.1879
	2	121 (30)			116 (28)	−4 (−8 to 1)	0.1245		—

Abbreviations: diff=difference; TO = baseline; T1 = week 6, midway; T2 = week 12, completed; T3 = week 39, follow-up; n = maximum n for patients at baseline test and associated follow-up test; *=between-group significance p < 0.05; SD = standard deviation; CI = confidence interval; diff = difference; 1RM = one repetition maximum test; allocation group 1 = supervised exercise intervention; allocation group 2 = instructed pedometer intervention.

#### Body composition

Body weight remained stable during the intervention period, with a significant decrease at T3 in the supervised exercise intervention, though there were no between-group differences. Lean body mass increased significantly from T0-T2 and dropped to baseline levels at T3. Fat mass decreased in the supervised exercise intervention at follow-up (T3) with no difference observed between groups. In both groups there was a significant loss of bone mass content from T0 to T3, while bone mass density had increased significantly in both study groups at T3. T-scores decreased significantly from T0 to T3 without reaching levels for osteopenia^50^. (Table 4).

**Table 4:** DXA scan body mass measures.

Variable	Group	T0: Mean (SD) (n = 153)	T2: Mean (SD) (n = 128)	T3: Mean (SD) (n = 115)	Delta (95% CI)	p	diff (95% CI)	p
Weight (kg)	1	74.7 (14.3)	74.6 (13.9)		−0.1 (−1 to 0.8)	0.8320	−0.4	0.5866
	2	73 (13.7)	73.3 (14)		0.3 (−0.7 to 1.2)	0.576	(−1.7 to 1.0)	
	1	75.1 (14.7)		74 (13.6)	−1.1 (−2.1 to −0.2)	0.0215	−0.6	0.4068
	2	72.7 (13)		72.1 (13.2)	−0.6 (−1.5 to 0.4)	0.2594	(−2.0 to 0.8)	
Fat mass (g)	1	29342 (10039)	28169 (9815)		−1152 (−2307 to 3)	0.0507	−834 (−2457 to 788)	0.3120
	2	28060 (9358)	27767 (9601)		−318 (−1457 to 822)	0.5834		
	1	29859 (10179)		30269 (11690)	456 (−736 to 1648)	0.4522	795 (−894 to 2484)	0.3548
	2	27678 (8754)		27353 (8707)	−339 (−1536 to 858)	0.5770		
Bone mass content (g)	1	2497.2 (346.2)	2492.5 (350.8)		−4.2 (−22.8 to 14.5)	0.6608	−13.3	0.3191
	2	2407.9 (337.9)	2417.2 (333.2)		9.1 (−9.3 to 27.5)	0.3299	(−39.4 to 12.9)	
	1	2482.7 (346.4)		2433.1 (335.5)	−48.4 (−67.6 to −29.1)	<0.0001	−12.9	0.3513
	2	2412.3 (348.6)		2376.5 (344.6)	−35.4 (−54.8 to −16.1)	0.0004	(−40.2 to 14.3)	
Lean body mass (g)	1	42842 (5218)	43916 (5304)		1048 (592 to 1505)	<0.0001	486 (−156 to 1127)	0.1372
	2	42545 (5465)	43102 (5710)		563 (112 to 1013)	0.0146		
	1	42733 (5380)		42722 (5057)	−78 (−549 to 394)	0.7456	82 (−586 to 750)	0.8086
	2	42556 (5402)		42442 (5398)	−160 (−633 to 314)	0.5064		
Bone mineral density	1	1.20 (0.12)	1.23 (0.23)		0.03 (0.00 to 0.07)	0.0696	0.02 (−0.03 to 0.07)	0.459
	2	1.16 (0.19)	1.18 (0.12)		0.01 (−0.02 to 0.05)	0.4283		
	1	1.19 (0.11)		1.18 (0.11)	−0.01 (−0.04 to 0.03)	0.6288	0.00 (−0.05 to 0.05)	0.9815
	2	1.16 (0.19)		1.15 (0.12)	−0.01 (−0.04 to 0.03)	0.6066		
T-score (SD)	1	1.21 (1.19)	1.19 (1.18)		−0.02 (−0.08 to 0.05)	0.6585	−0.04 (−0.13 to 0.06)	0.4343
	2	0.93 (1.24)	0.95 (1.18)		0.02 (−0.04 to 0.09)	0.5056		
	1	1.14 (1.15)		0.99 (1.10)	−0.15 (−0.22 to −0.08)	<0.0001	0.03 (−0.07 to 0.13)	0.5395
	2	0.86 (1.24)		0.69 (1.21)	−0.18 (−0.25 to −0.11)	<0.0001		

Abbreviations: TO = baseline; T1 = week 6, midway; T2 = week 12, completed; T3 = week 39, follow-up; DXA = dual-energy X-ray absorptiometry; n = maximum n for patients at baseline test and associated follow-up test; 1 = group 1 supervised exercise intervention; 2 = group 2 pedometer exercise intervention; SD = standard deviation; CI = confidence interval.

### Blood markers

Blood markers showed no between-group difference (P-glucose, P-insulin, P-cholesterol, high-density lipoprotein cholesterol, high-density lipoprotein cholesterol), except for P-triglyceride, which was significantly lowered in the pedometer group at week 39 (T3). Total cholesterol generally remained elevated (5.3–5.4 SD 1.1) and unchanged. Blood glucose and cholesterol remained relatively stable, though a slightly significant increase was visible for high-density lipoprotein in Gr. 2 at follow-up (T3). For a full analysis see online Supplemental Material.

**Figure 5:**
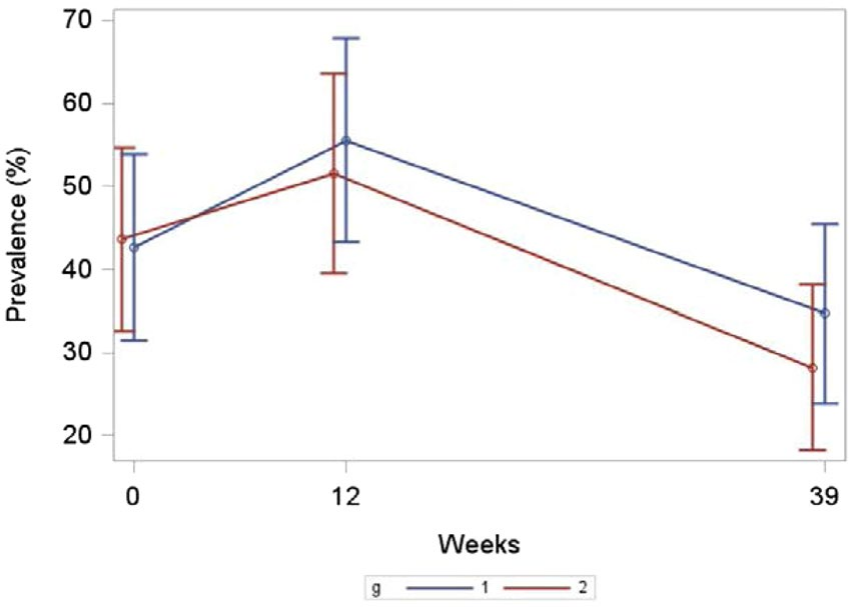
Metabolic syndrome.

A composite metabolic syndrome score of five biological and physiological variables was calculated for each group (Fig. 5). Three (or more) out of five risk variables were classified as a case. The findings revealed a significant improvement in the metabolic risk profile from baseline to week 39 for both groups (Gr. 1, *p* = 0.0493; Gr. 2, *p* = 0.0156).

### Selected patient-reported outcomes

There were no significant between-group changes on the European Organisation for Research and Treatment of Cancer Quality of Life Questionnaire (EORTC QLQ) or the Hospital Anxiety Depression Scale. Fatigue, pain, dyspnoea and insomnia worsened significantly during chemotherapy without between-group differences and was restored significantly to baseline levels at follow-up. Anxiety remained relatively stable during the study period, whereas depression increased from baseline to T2 and was significantly below the baseline values at T3 (Table 5).

**Table 5:** Selected patient-reported outcomes.

Panel A:	Group	T0 (n = 153)	T1 (n = 125)	T2 (n = 126)	T3 (n = 117)	(95% CI)	p Value	Diff.	p
HADS		Mean (SD)	Mean (SD)	Mean (SD)	Mean (SD)			(95% CI)	Value
HADS Anxiety	1	4.4 (4.0)	4.4 (3.9)			1.0 (0.4 to 1.7)	0.002	0.9 (0.0 to 1.8)	0.0591
(Scale 0–21)	2	5.2 (3.8)	4.8 (3.9)			0.2 (−0.5 to 0.8)	0.6483		
	1	4.4 (4)		4.0 (3.7)		1.3 (0.5 to 2.1)	0.0017	−0.3 (−1.4 to 0.8)	0.6251
	2	5.2 (3.9)		5.3 (3.9)		1.5 (0.8 to 2.3)	0.0001		
	1	4.3 (4.0)			3.9 (3.7)	−0.8 (−1.5 to −0.1)	0.0304	−0.3 (−1.4 to 0.7)	0.5193
	2	5.0 (3.8)			4.8 (4.0)	−0.5 (−1.2 to 0.3)	0.2108		
HADS Depression	1	3.3 (2.8)	4.3 (3.3)			1.0 (0.4 to 1.7)	0.002	0.9 (0 to 1.8)	0.0591
(Scale 0–21)	2	3.7 (3.5)	4.0 (3.7)			0.2 (−0.5 to 0.8)	0.6483		
	1	3.3 (2.8)		4.6 (3.4)		1.3 (0.5 to 2.1)	0.0017	−0.3 (−1.4 to 0.8)	0.6251
	2	3.7 (3.4)		5.4 (4.0)		1.5 (0.8 to 2.3)	0.0001		
	1	3.2 (2.8)			2.5 (2.7)	−0.8 (−1.5 to −0.1)	0.0304	−0.3 (−1.4 to 0.7)	0.5193
	2	3.4 (3.2)			3.1 (3.1)	−0.5 (−1.2 to 0.3)	0.2108		
**Panel B: Selected EORTC scales**									
EORTC Global Health Status	1	60 (21)	52 (24)			−8 (−14 to −3)	0.0021	−6 (−14 to 1)	0.1125
(Scale 0–100)	2	60 (21)	58 (23)			−2 (−8 to 3)	0.3780		
	1	61 (21)		56 (22)		−4 (−10 to 2)	0.1905	6 (−2 to 15)	0.1288
	2	62 (22)		51 (20)		−11 (−16 to −5)	0.0005		
	1	62 (21)			76 (19)	15 (9 to 21)	<0.0001	8 (0 to 16)	0.0517
	2	63 (21)			69 (23)	7 (1 to 13)	0.0208		
EORTC Fatigue	1	42 (25)	52 (26)			10 (3 to 17)	0.0036	8 (−2 to 18)	0.0977
(Scale 0–100)	2	49 (25)	50 (26)			2 (−5 to 9)	0.5333		
	1	42 (25)		58 (27)		17 (9 to 25)	<0.0001	5 (−6 to 16)	0.3491
	2	48 (25)		59 (25)		12 (4 to 19)	0.0033		
	1	40 (25)			29 (24)	−12 (−18 to −5)	0.0005	−4 (−13 to 6)	0.4300
	2	46 (24)			38 (26)	−8 (−15 to −1)	0.0168		
	1	17 (21)	31 (32)			15 (7 to 24)	0.0004	9 (−3 to 21)	0.1357
EORTC Pain	2	22 (26)	29 (31)			6 (−2 to 15)	0.1236		
(Scale 0–100)	1	18 (21)		32 (30)		16 (8 to 24)	<0.0001	−3 (−14 to 8)	0.6209
	2	22 (26)		42 (29)		19 (11 to 27)	<0.0001		
	1	17 (20)			22 (22)	6 (−1 to 12)	0.0778	6 (−3 to 15)	0.1973
	2	20 (24)			21 (23)	0 (−7 to 6)	0.9464		
	1	10 (22)	20 (23)			11 (4 to 17)	0.0026	4 (−5 to 14)	0.3656
EORTC Dyspnoea	2	13 (22)	19 (27)			6 (−1 to 13)	0.0707		
(Scale 0–100)	1	10 (22)		28 (31)		18 (9 to 27)	0.0001	2 (−11 to 14)	0.7901
	2	12 (22)		30 (31)		16 (8 to 25)	0.0003		
	1	10 (22)			10 (19)	0 (−7 to 7)	0.9566	0 (−10 to 9)	0.9885
	2	8 (17)			12 (22)	0 (−6 to 7)	0.9407		

Abbreviations: *=between-group difference; TO = baseline; T1 = week 6, midway; T2 = week 12, completed; T3 = week 39, follow-up; 1 = group 1 supervised exercise intervention; 2 = group 2 pedometer exercise intervention; SD = standard deviation; CI = confidence interval; EORTC = European Organisation for Research and Treatment of Cancer − Quality of Life Questionnaire; HADS = Hospital Anxiety Depression Scale.

**Figure 6:**
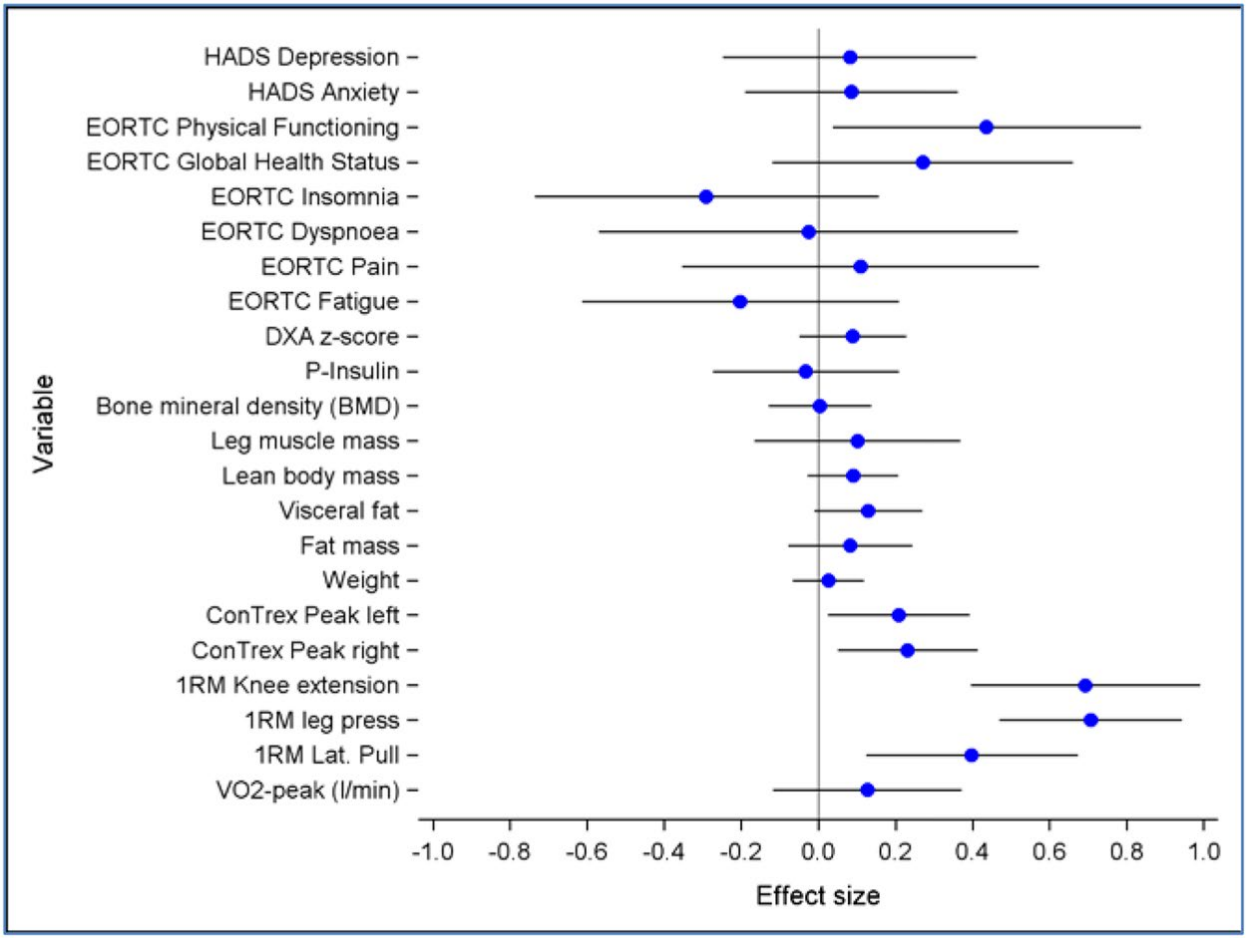
Global overview of effect sizes.

Figure 6 provides an overview and comparison of effect sizes (cohens *d*) between study groups at T2 assessment for the primary outcome, VO_2_peak, and secondary measures. In general, effect sizes reflect benefits favouring the supervised exercise intervention in physiological outcomes and physical functioning, whereas effects on patient-reported measures were heterogenous between groups.

#### Explorative analysis of VO_2_ peak

By merging VO_2_peak data across study groups, we dichotomised VO_2_peak into positive responders/unchanged (n = 41, 32%) and negative responders >2% (n = 87, 68%) from baseline to week 12. Positive/unchanged responders had improved clinical effects on EORTC QLQ scores versus negative responders: fatigue (51.3 SD 28 vs. 60.0 SD 24.6); pain (26.9 SD 25.8 vs. 41.7 SD 30.7); and dyspnoea (17.9 SD 26.3 vs. 30.7 SD 28.9). There were no significant differences in baseline scores or at follow-up (T3) between responders and decliners.

Correlation analyses between changes in (delta), physiological outcomes (VO_2_peak, muscle strength) and (delta) selected patient-reported outcome scales (fatigue, pain, dyspnoea) at week 12 revealed a very weak correlation between strength and delta fatigue, pain and dyspnoea, whereas VO_2_peak demonstrated a significant weak correlation on EORTC QLQ fatigue, pain and dyspnoea. Parametric testing indicated that an improvement on VO_2_peak with 100 ml/min would decrease fatigue by 4 points, whereas, e.g. a gain in lean body mass by 0.5 kg would result in a decrease in fatigue by 1.4 points. Delta VO_2_peak (100 ml) or delta leg press (10 kg) improvements were significantly correlated with reduced pain scores (average 3 EORTC QLQ points). (Table 6).

**Table 6:** Correlations and parametric values between delta physiological outcomes and delta selected patient-reported outcome scales.

Panel A: Correlation	Δ Fatigue	Δ Pain	Δ Dyspnoea
Δ Contrex right	−0.12 (−0.29, 0.06)	−0.01 (−0.19, 0.16)	0.04 (−0.14, 0.22)
Δ Contrex left	−0.04 (−0.22, 0.13)	−0.03 (−0.21, 0.14)	−0.04 (−0.22, 0.13)
Δ Lean mass	−0.18 (−0.34, 0.00)	−0.11 (−0.28, 0.07)	−0.04 (−0.21, 0.14)
Δ Leg press	−0.07 (−0.25, 0.11)	−0.22 (−0.38, −0.04)	−0.12 (−0.29, 0.07)
Δ VO_2_peak	−0.31 (−0.46, −0.14)	−0.25 (−0.41, −0.08)	−0.27 (−0.42, −0.09)
**Panel B: Parametric**			
Δ Contrex right (5 N m)	−1.3 (−3.3, 0.7)	−0.1 (−2.1, 1.8)	0.5 (−1.7, 2.7)
Δ Contrex left (5 N m)	−0.5 (−2.4, 1.4)	−0.4 (−2.3, 1.5)	−0.5 (−2.6, 1.6)
Δ Lean Mass (0.5 kg)	−1.4 (−2.8, 0.0)	−0.8 (−2.3, 0.6)	−0.3 (−2.0, 1.3)
Δ Leg press (10 kg)	−0.8 (−3.2, 1.7)	−2.7 (−5.2, −0.3)	−1.8 (−4.5, 1.0)
Δ VO_2_peak (100 ml)	−4.0 (−6.1, −1.8)	−3.2 (−5.4, −1.1)	−3.7 (−6.1, −1.3)

## Discussion

Contrary to our a priori hypothesis, we observed no in-between group difference on the primary outcome, VO_2_peak (CRF), in supervised hospital-based moderate to high intensity group exercise intervention versus an instructed home-based individual pedometer programme. A decline in VO_2_peak was observed in both groups after onset of chemotherapy, and VO_2_peak was fully restored at week 39. Our results disprove a lasting CRF loss due to chemotherapy, as previously suggested^14–16^. Other intervention RCTs have found an even further reduction in CRF during adjuvant taxane-based chemotherapy, though some natural improvement in CRF may occur in RCT controls^23,31,32^. In observational studies CRF has been inversely associated with mortality in clinical and population cohorts^11,51–54^. Furthermore, physically inactive survivors of BC are at increased risk of post-treatment cardiovascular disease and cancer recurrence^29,55,56^, and only few powered intervention RCTs have explored the adaptationally positive impact of regular PA on disease-free survival and all-cause mortality^57^. There is also a lack of studies explicitly recruiting cancer patients to PA interventions during treatment who were physically inactive/sedentary prediagnosis^26,58^. Consequently, we cannot clarify whether results from the vast majority of studies and meta-analyses are derived from selective populations of cancer patients with a predominant preference for PA^21,59–61^, or whether recruitment, intervention activities and outcome expectations can be transferred to an at-risk subgroup with less PA experience and beyond the context of a trial environment. Patient-reported PA levels, a secondary outcome, supported our physiological findings, and we suggest that a loss in CRF can be restored by sustainable adaption to national PA guidelines across intervention approaches and among physically inactive survivors with BC.

The two-item screening tool based on national PA guidelines identified 60% as being physically inactive against 40% meeting PA guidelines at chemotherapy onset. The study acceptance rate of 45% is comparable to exercise RCTs that do not exclusively recruit screened, physically inactive patients with BC referred to adjuvant chemotherapy^32,62^. The randomly considered study attrition rate was acceptable for both groups. In addition to the level of physical inactivity identified, we observed several physiological and biological indicators of metabolic syndrome that were raised among 42% of the recruited population, constituting an elevated risk profile. At baseline, 23 patients (15%) were medically treated for a cardiovascular risk component and were moderately overweight, with an average body mass index >26 (SD 5.15]. Several studies have observed weight gain following BC treatment associated with an increased metabolic long-term risk^63–65^. Metabolic syndrome may increase BC recurrence three-fold and BC-specific mortality approximately two-fold^66^. CRF and muscle strength have been shown to have an inversely independent association on metabolic cardiovascular risk factors in adults^67^. Likewise, moderate to vigorous leisure time PA is inversely associated with metabolic syndrome^68^. Thus, the improved metabolic risk profile and stable weight development achieved in the present study validate the cohesion of physiological and patient-reported PA outcomes, suggesting the treatment complementary effectiveness of the interventions to improve important clinical risk outcomes. We believe this transverse cohesion, along with the systematic recruitment of physically inactive BC patients, constitutes the primary clinical importance in the approach and findings of this trial.

In line with several RCTs and metanalysis evaluating combined cardio and resistance exercise interventions^31,32,69,70^, our findings indicate a significant positive effect on physical functioning in the supervised exercise group, with an effect size considered subjectively meaningful^71^. Similarly, in agreement with previous studies evaluating the effect of resistance exercise^19,23,31,32,72^, we found that participation in the supervised exercise intervention maintained or improved muscle strength in the lower and upper extremities, with one repetition maximum strength increases corresponding to 20% and 22% (knee extension and leg press, respectively) and 17% in the lateral pull exercise post-intervention. Muscle strength after chemotherapy for BC survivors without intervention has been found to be 12–16% lower in the upper extremities, and 25% lower in the lower extremities, compared to healthy women^72^. This is of potential clinical importance as declines in muscle strength have been associated with loss of function in BC survivors, affecting the ability to perform recreational and daily living activities^73^. Considering the relationship between muscle strength and sarcopenia, our findings have potential clinical implications as BC sarcopenia, even early stage, has been associated with poorer survival and shorter time to tumour progression^74^. Our results indicate that exercise during adjuvant chemotherapy, with supervised heavy-load resistance training, provides the strongest effects and may ameliorate the detrimental effects of chemotherapy on skeletal muscles^72,74,75^, irrespective of cardiorespiratory deterioration.

EORTC patient-reported outcomes showed an increasing symptom profile on, e.g. fatigue, pain, dyspnoea and insomnia during the intervention period and without any in-between group differences. However, the explorative analyses showed that, across groups, the individuals who were able to maintain or improve CRF (33%) showed significant improvement on several EORTC QLQ scales. The correlation analyses between physiological outcomes and patient-reported outcomes suggested that CRF is inversely correlated with reduced fatigue, pain and dyspnoea, while muscle indicators had almost no correlation. This finding is in line with the study’s rationale and overall assumptions^38^. These results support the notion that pathways and interplay between physiological measures and patient perceived benefits on symptomatology and side effects are complex and far from being understood^31^.

The two present interventions were designed to appeal to and motivate BC patients who did not meet the Danish national PA guidelines and were concurrently referred to adjuvant chemotherapy. The findings showed that interventions were equally effective with respect to supporting behavioural changes towards adopting an adequate transition to PA in everyday life activities for several participants, during and post chemotherapy. It has been suggested that health professional counselling rooted in cognitive behavioural therapy and possessing a central basis in PA interventions has a positive effect on maintaining PA adherence rate for patients with cancer^76^. Two studies preceding the present RCT indicated that recommendations from the oncologist, the screening procedure, the objective physiological tests and the counselling sessions with the clinical nurse specialist were crucial to patient recruitment, adherence and patients feeling safe when initiating PA during their treatment cycles^27,36^. The recurring counselling sessions were carried out face to face between the clinical nurse specialist and patients. Besides serving as motivation, these sessions, by incorporating social cognitive theory^77^ and behavioural change strategies^36,78^, targeted exercise intolerance by taking into account the individual patient’s symptom profile, reducing side effect-related dropout and by addressing the habits and structural barriers in daily living. Accordingly, we suggest that interventions should be integrated into a treatment context that aligns with patient needs and preferences in terms of becoming physically active during adjuvant chemotherapy^36^. Consequently, exercise oncology ideology based primarily on physiological mechanisms and exercise prescriptions^79^ may potentially underestimate the role the human dynamic plays, as well as the environmental impact on motivation and PA sustainability^36^.

One of the strengths in the present study is the focus on a longitudinal clinical profile in PA screened survivors with breast cancer by using gold standard measures on various physiological outcomes with a low risk of bias. The clinicians who pre-screened for physical inactivity present at adjuvant chemotherapy secured recruitment of the targeted population at risk, underpinning the validity and reliability of the two-item screening tool. A potential selection bias relates to the fact that patients were likely to be more highly educated. This is consistent, on the one hand, with the inverse social gradient in BC populations^80,81^, but could indicate, on the other, selection of the most highly motivated among the less active. It is a weakness that we did not incorporate a non-intervention control group, but this was not possible for ethical reasons associated with standard hospital exercise programmes^19^. The 15% dropout rate at T2 may have caused a selection bias, though we did not identify explanatory variables significantly associated with the attrition.

## Conclusions

In conclusion, our study shows that beneficial effects can be obtained from supervised intensive, hospital-based exercise programmes but also from a less demanding pedometer exercise intervention under guidance and counselling from committed health professionals. Both interventions were effective in supporting formerly inactive BC patients in sustaining PA activities during and following adjuvant treatment. Restored CRF at follow-up and metabolic indicators showed an improved health profile and subsequently a decline in cardiovascular risk. Clinicians should address patient-specific modifiable risks within a medical and preventative scope, especially to support motivational progress and to counteract cumbersome barriers, such as treatment-related toxicity, factors that constantly challenge motivation and exercise intolerance in physically inactive BC survivors.

## Supplementary information

Supplemental file.

CONSORT list.

## Data Availability

Anonymous data are available upon from the corresponding author (TM) upon reasonable request. The datasets generated and/or analysed during the current study are available from the corresponding author upon reasonable request.
