# Supplementary material for: Physical deterioration and adaptive recovery in physically inactive breast cancer patients during adjuvant chemotherapy: a randomised controlled trial
Source: Sci Rep. 2020 Jun 16;10:9710. doi: 10.1038/s41598-020-66513-9 (PMC7297957; doi:10.1038/s41598-020-66513-9)
Supplement: Supplementary file 1 — Supplemental file. [file 41598_2020_66513_MOESM1_ESM.pdf]

Title:

Physical deterioration and adaptive recovery in physically inactive breast cancer patients during adjuvant chemotherapy: a randomised controlled trial

Author block

Tom Møller (RN, MPH, PhD), Christina Andersen (RN, MPH PhD), Christian Lillelund (physiotherapist, MSc), Kira Bloomquist (physiotherapist, MSc, PhD), Karl Bang Christensen (MSc, PhD), Bent Ejlertsen (MD, PhD), Malgorzata Tuxen (MD PhD), Peter Oturai (MD), Ulla Breitenstein (RN), Cecilie Kolind (RN), Pernille Travis (RN), Tina Bjerg (RN), Mikael Rørth (MD) and Lis Adamsen, (RN, MSocSc, PhD)

Supplementary file: EORTC-QLQ-C30

variable	gr	n	Mean (SD)				Δ (95% CI)	P	Diff. (95% CI)	P
			0	6	12	39				
EORTC Global Health Status	1	60	60 (21)	52 (24)			-8 (-14 to -3)	0.0021	-6 (-14 to 1)	0.1125
		12	62 (19)							
	2	60	60 (21)	58 (23)			-2 (-8 to 3)	0.3780		
		16	67 (21)							
	1	59	61 (21)		56 (22)		-4 (-10 to 2)	0.1905	6 (-2 to 15)	0.1288
		13	56 (17)							
	2	63	62 (22)		51 (20)		-11 (-16 to -5)	0.0005		
		13	62 (20)							
	1	59	62 (21)			76 (19)	15 (9 to 21)	<.0001	8 (0 to 16)	0.0517
		13	51 (19)							
	2	55	63 (21)			69 (23)	7 (1 to 13)	0.0208		
		21	58 (23)							
EORTC Physical Functioning	1	60	87 (12)	88 (12)			0 (-3 to 3)	0.8382	1 (-3 to 5)	0.5762
		12	90 (7)							
	2	60	87 (13)	86 (13)			-2 (-4 to 1)	0.3167		
		16	92 (10)							
	1	59	87 (12)		89 (12)		1 (-2 to 5)	0.5170	6 (1 to 11)	0.0175
		13	90 (8)							
	2	63	87 (13)		82 (16)		-5 (-8 to -1)	0.0062		
		13	91 (8)							
	1	59	88 (12)			91 (13)	4 (0 to 7)	0.0297	4 (-1 to 8)	0.1303
		13	87 (7)							
	2	55	89 (12)			88 (13)	0 (-3 to 3)	0.9777		
		21	85 (15)							
EORTC Role Functioning	1	60	67 (30)	64 (29)			-6 (-14 to 2)	0.1565	-5 (-17 to 6)	0.3436
		12	88 (20)							
	2	60	62 (30)	63 (31)			0 (-8 to 8)	0.9313		
		16	74 (29)							
	1	59	68 (30)		61 (28)		-9 (-17 to -1)	0.0297	-3 (-14 to 8)	0.5857

		13	81 (24)							
	2	63	62 (30)		58 (26)		-6 (-13 to 2)	0.1411		
		13	76 (28)							
	1	59	71 (28)			84 (19)	13 (5 to 21)	0.0016	0 (-11 to 11)	0.9816
		13	71 (34)							
	2	55	65 (29)			78 (30)	13 (5 to 21)	0.0016		
		21	63 (32)							
EORTC Emotional Functionin	1	60	79 (21)	80 (21)			1 (-3 to 5)	0.6746	-4 (-10 to 2)	0.2192
		12	77 (20)							
	2	60	73 (20)	78 (21)			5 (0 to 9)	0.0316		
		16	67 (24)							
	1	59	79 (21)		78 (24)		-1 (-6 to 3)	0.6060	-4 (-10 to 3)	0.2524
		13	79 (21)							
	2	63	74 (20)		76 (20)		2 (-2 to 7)	0.2656		
		13	62 (21)							
	1	59	79 (21)			82 (21)	3 (-2 to 7)	0.2806	-1 (-8 to 5)	0.6640
		13	76 (20)							
	2	55	74 (20)			78 (21)	4 (-1 to 9)	0.0981		
		21	66 (23)							
EORTC Cognitive functionin	1	60	78 (21)	68 (26)			-10 (-16 to -5)	0.0004	-9 (-17 to -1)	0.0247
		12	81 (22)							
	2	60	73 (21)	71 (28)			-1 (-7 to 4)	0.6681		
		16	65 (30)							
	1	59	78 (22)		71 (22)		-7 (-13 to -2)	0.0077	-3 (-10 to 5)	0.4751
		13	79 (21)							
	2	63	73 (21)		67 (25)		-5 (-10 to 1)	0.0804		
		13	65 (32)							
	1	59	77 (22)			76 (23)	-2 (-7 to 4)	0.5244	-4 (-12 to 4)	0.3614
		13	81 (21)							
	2	55	75 (19)			76 (22)	2 (-4 to 8)	0.5123		
		21	62 (31)							
	1	60	76 (23)	71 (23)			-6 (-12 to 0)	0.0356	-4 (-12 to 4)	0.2725
		12	88 (16)							

EORTC Social Functioning	2	60	78 (24)	76 (26)			-2 (-7 to 4)	0.5653		
		16	80 (24)							
	1	59	76 (24)		68 (24)		-9 (-15 to -3)	0.0055	1 (-8 to 10)	0.7877
		13	85 (16)							
	2	63	79 (23)		68 (28)		-10 (-16 to -4)	0.0013		
		13	76 (24)							
	1	59	77 (23)			86 (22)	9 (2 to 15)	0.0078	2 (-7 to 11)	0.6490
		13	78 (21)							
	2	55	82 (20)			86 (18)	7 (0 to 13)	0.0435		
		21	69 (29)							
EORTC Fatique	1	60	42 (25)	52 (26)			10 (3 to 17)	0.0036	8 (-2 to 18)	0.0977
		12	39 (28)							
	2	60	49 (25)	50 (26)			2 (-5 to 9)	0.5333		
		16	40 (32)							
	1	59	42 (25)		58 (27)		17 (9 to 25)	<.0001	5 (-6 to 16)	0.3491
		13	41 (28)							
	2	63	48 (25)		59 (25)		12 (4 to 19)	0.0033		
		13	44 (32)							
	1	59	40 (25)			29 (24)	-12 (-18 to -5)	0.0005	-4 (-13 to 6)	0.4300
		13	51 (29)							
	2	55	46 (24)			38 (26)	-8 (-15 to -1)	0.0168		
		21	50 (33)							
EORTC Nausea and vomiting	1	60	17 (22)	10 (16)			-6 (-12 to 0)	0.0353	-4 (-12 to 4)	0.3212
		11	12 (21)							
	2	60	18 (20)	16 (20)			-2 (-8 to 4)	0.4610		
		16	19 (23)							
	1	59	16 (23)		6 (12)		-10 (-15 to -4)	0.0008	3 (-5 to 11)	0.4205
		12	14 (20)							
	2	63	17 (20)		5 (16)		-13 (-18 to -7)	<.0001		
		13	23 (24)							
	1	59	16 (23)			2 (7)	-14 (-19 to -9)	<.0001	0 (-7 to 7)	0.9428
		12	15 (19)							
	2	55	15 (17)			4 (9)	-14 (-19 to -9)	<.0001		

		21	25 (27)							
EORTC Pain	1	60	17 (21)	31 (32)			15 (7 to 24)	0.0004	9 (-3 to 21)	0.1357
		12	10 (15)							
	2	60	22 (26)	29 (31)			6 (-2 to 15)	0.1236		
		16	26 (31)							
	1	59	18 (21)		32 (30)		16 (8 to 24)	<.0001	-3 (-14 to 8)	0.6209
		13	9 (15)							
	2	63	22 (26)		42 (29)		19 (11 to 27)	<.0001		
		13	28 (31)							
	1	59	17 (20)			22 (22)	6 (-1 to 12)	0.0778	6 (-3 to 15)	0.1973
		13	13 (22)							
	2	55	20 (24)			21 (23)	0 (-7 to 6)	0.9464		
		21	30 (31)							
EORTC Dyspnoea	1	59	10 (22)	20 (23)			11 (4 to 17)	0.0026	4 (-5 to 14)	0.3656
		12	8 (15)							
	2	60	13 (22)	19 (27)			6 (-1 to 13)	0.0707		
		16	17 (30)							
	1	58	10 (22)		28 (31)		18 (9 to 27)	0.0001	2 (-11 to 14)	0.7901
		13	8 (15)							
	2	63	12 (22)		30 (31)		16 (8 to 25)	0.0003		
		13	21 (32)							
	1	58	10 (22)			10 (19)	0 (-7 to 7)	0.9566	0 (-10 to 9)	0.9885
		13	8 (15)							
	2	55	8 (17)			12 (22)	0 (-6 to 7)	0.9407		
		21	27 (33)							
EORTC Insomnia	1	60	23 (31)	36 (36)			12 (4 to 20)	0.0049	13 (1 to 24)	0.0324
		12	28 (28)							
	2	60	33 (29)	32 (31)			-1 (-9 to 7)	0.8519		
		16	29 (21)							
	1	59	24 (32)		45 (36)		21 (12 to 31)	<.0001	8 (-5 to 21)	0.2199
		13	26 (28)							
	2	63	33 (29)		46 (32)		13 (4 to 22)	0.0050		
		13	28 (18)							

	1	59	24 (32)			31 (36)	6 (-3 to 15)	0.1834	2 (-12 to 15)	0.8132
		13	26 (28)							
	2	55	33 (30)			36 (36)	5 (-5 to 14)	0.3296		
		21	32 (20)							
EORTC Appetite loss	1	60	18 (25)	20 (29)			2 (-5 to 10)	0.5686	-3 (-13 to 8)	0.6144
		12	14 (17)							
	2	60	20 (28)	25 (30)			5 (-3 to 12)	0.1988		
		16	21 (30)							
	1	59	19 (25)		19 (27)		1 (-7 to 10)	0.7407	-1 (-13 to 11)	0.9076
		13	13 (17)							
	2	63	20 (27)		22 (30)		2 (-6 to 11)	0.6117		
		13	23 (32)							
	1	59	19 (25)			6 (18)	-12 (-19 to -6)	0.0002	2 (-7 to 11)	0.6556
		13	10 (16)							
	2	55	19 (27)			5 (14)	-14 (-21 to -8)	<.0001		
		21	24 (30)							
EORTC Constipation	1	60	21 (26)	16 (23)			-4 (-11 to 3)	0.2805	3 (-7 to 13)	0.5727
		12	8 (21)							
	2	60	26 (29)	18 (24)			-7 (-14 to 0)	0.0598		
		16	23 (23)							
	1	59	20 (26)		16 (25)		-3 (-10 to 4)	0.3347	4 (-5 to 14)	0.3692
		13	13 (22)							
	2	63	25 (29)		17 (26)		-8 (-15 to -1)	0.0235		
		13	28 (23)							
	1	59	21 (26)			5 (14)	-14 (-21 to -7)	<.0001	2 (-8 to 11)	0.6956
		13	10 (21)							
	2	55	25 (27)			10 (22)	-16 (-23 to -9)	<.0001		
		21	25 (30)							
EORTC Diarrhoea	1	60	12 (24)	22 (28)			10 (3 to 16)	0.0039	4 (-5 to 13)	0.4235
		12	14 (17)							
	2	60	13 (25)	19 (26)			6 (-1 to 12)	0.0717		
		15	18 (28)							
	1	59	11 (24)		19 (26)		7 (-1 to 14)	0.0959	-6 (-17 to 5)	0.2631

		13	15 (17)							
	2	63	12 (25)		26 (28)		13 (5 to 20)	0.0011		
		12	22 (30)							
	1	59	12 (24)			7 (19)	-5 (-13 to 3)	0.2059	0 (-11 to 10)	0.9306
		13	13 (17)							
	2	55	8 (17)			9 (22)	-4 (-12 to 3)	0.2551		
		20	30 (37)							
EORTC Financial difficulti	1	60	6 (21)	6 (21)			1 (-3 to 4)	0.7714	1 (-4 to 6)	0.8161
		12	6 (13)							
	2	60	6 (18)	7 (19)			0 (-4 to 4)	0.9702		
		16	15 (24)							
	1	59	6 (22)		8 (23)		3 (-1 to 7)	0.1452	3 (-3 to 8)	0.3262
		13	5 (13)							
	2	63	6 (17)		6 (19)		0 (-3 to 4)	0.9262		
		13	18 (26)							
	1	59	6 (22)			6 (21)	1 (-4 to 6)	0.7304	3 (-4 to 11)	0.3852
		13	5 (13)							
	2	55	7 (19)			4 (13)	-2 (-8 to 3)	0.3769		
		21	11 (22)							

Title:

Physical deterioration and adaptive recovery in physically inactive breast cancer patients during adjuvant chemotherapy: a randomised controlled trial

Author block

Tom Møller (RN, MPH, PhD), Christina Andersen (RN, MPH PhD), Christian Lillelund (physiotherapist, MSc), Kira Bloomquist (physiotherapist, MSc, PhD), Karl Bang Christensen (MSc, PhD), Bent Ejlertsen (MD, PhD), Malgorzata Tuxen (MD PhD), Peter Oturai (MD), Ulla Breitenstein (RN), Cecilie Kolind (RN), Pernille Travis (RN), Tina Bjerg (RN), Mikael Rørth (MD) and Lis Adamsen, (RN, MSocSc, PhD)

Supplementary file: Blood markers

variable		n	Mean (SD)			Δ (95% CI)	P	Diff. (95% CI)	P
			0	12	39				
P-Glukose	1	60	5.6 (0.9)	5.7 (1.1)		0.1 (-0.1 to 0.3)	0.1954	0.0 (-0.3 to 0.3)	0.9157
	1	14	5.5 (1.6)						
	2	61	5.5 (0.8)	5.6 (0.9)		0.1 (-0.1 to 0.3)	0.2469		
	2	15	5.1 (0.4)						
	1	56	5.6 (0.9)		5.6 (1.1)	0.0 (-0.2 to 0.2)	0.9453	0.0 (-0.3 to 0.3)	0.8663
	1	18	5.6 (1.4)						
	2	56	5.4 (0.8)		5.4 (1.0)	0.0 (-0.2 to 0.2)	0.7585		
	2	20	5.4 (0.7)						
P-Insulin	1	57	75.1 (51.5)	70.4 (50.4)		-5.3 (-15.3 to 4.6)	0.2923	1.9 (-12.3 to 16.1)	0.7893
	1	16	75.2 (53.7)						
	2	54	75.7 (68.4)	67.1 (61.4)		-7.3 (-17.4 to 2.9)	0.1587		
	2	18	70.2 (55.1)						
	1	52	75 (56.2)		67.6 (37.1)	-6.7 (-17 to 3.6)	0.2008	-2.1 (-16.7 to 12.5)	0.7764
	1	21	75.4 (39.2)						
	2	51	64.8 (34.1)		59.9 (27.7)	-4.6 (-14.9 to 5.7)	0.3808		
	2	21	97.6 (106.3)						
P-kolesterol	1	63	5.3 (1.1)	5.3 (1.1)		0.0 (-0.3 to 0.3)	0.9582	-0.2 (-0.6 to 0.2)	0.3753
	1	12	5.2 (1)						
	2	62	5.4 (1.1)	5.5 (1.7)		0.2 (-0.1 to 0.5)	0.2302		
	2	14	4.8 (1.1)						
	1	57	5.3 (1)		5.5 (1.1)	0.2 (-0.1 to 0.5)	0.1729	0.1 (-0.3 to 0.5)	0.6426
	1	18	5.1 (1.2)						
	2	56	5.4 (1.2)		5.4 (1.1)	0.1 (-0.2 to 0.4)	0.4794		
	2	20	4.9 (1.1)						
HDL kolesterol	1	63	1.6 (0.5)	1.3 (0.4)		-0.3 (-0.4 to -0.2)	<.0001	-0.1 (-0.2 to 0.1)	0.3218
	1	12	1.5 (0.4)						
	2	62	1.5 (0.4)	1.3 (0.3)		-0.3 (-0.4 to -0.2)	<.0001		
	2	14	1.5 (0.4)						
	1	57	1.6 (0.5)		1.7 (0.5)	0.1 (0.0 to 0.2)	0.0916	0.0 (-0.2 to 0.1)	0.4737
	1	18	1.6 (0.5)						
	2	56	1.6 (0.4)		1.7 (0.4)	0.1 (0.0 to 0.2)	0.0073		

	2	20	1.3 (0.3)						
LDL kolesterol	1	63	3.2 (1.0)	3.4 (0.9)		0.2 (0.0 to 0.4)	0.0474	0.0 (-0.3 to 0.3)	0.9627
	1	12	3.2 (0.9)						
	2	62	3.3 (1.0)	3.5 (1.0)		0.2 (0.0 to 0.4)	0.0560		
	2	13	2.9 (1.0)						
	1	57	3.3 (0.9)		3.4 (0.9)	0.2 (0.0 to 0.4)	0.0858	0.1 (-0.2 to 0.4)	0.4545
	1	18	3.1 (1.1)						
	2	56	3.3 (1.0)		3.4 (1.1)	0.1 (-0.1 to 0.3)	0.5089		
	2	19	3.1 (0.9)						
Triglycerid	1	63	1.1 (0.6)	1.4 (0.7)		0.3 (0.1 to 0.5)	0.0002	0.1 (-0.1 to 0.4)	0.2107
	1	12	1.5 (0.9)						
	2	62	1.4 (1.1)	1.5 (0.9)		0.2 (0.0 to 0.3)	0.0414		
	2	14	1.4 (1.2)						
	1	57	1.1 (0.7)		1.2 (0.6)	0.1 (-0.1 to 0.2)	0.3634	0.2 (0.0 to 0.5)	0.0466
	1	18	1.2 (0.8)						
	2	56	1.4 (1.2)		1.2 (0.7)	-0.2 (-0.3 to 0.0)	0.0563		
	2	20	1.4 (1.0)						
