# Supplementary material for: Physical deterioration and adaptive recovery in physically inactive breast cancer patients during adjuvant chemotherapy: a randomised controlled trial
Source: Sci Rep. 2020 Jun 16;10:9710. doi: 10.1038/s41598-020-66513-9 (PMC7297957; doi:10.1038/s41598-020-66513-9)
Supplement: Supplementary file 2 — CONSORT list. [file 41598_2020_66513_MOESM2_ESM.doc]

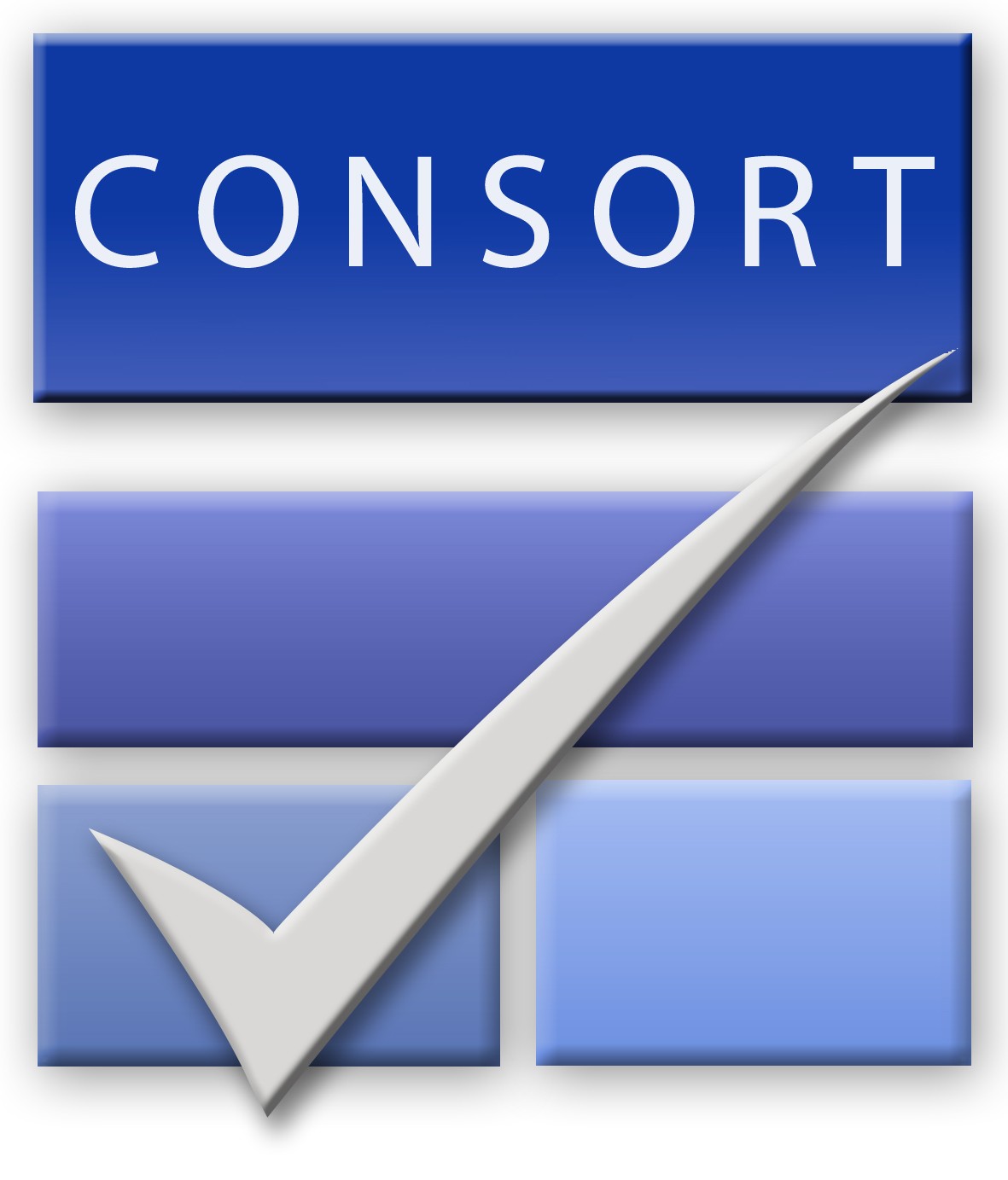
CONSORT 2010 checklist of information to include when reporting a randomised trial*

| Section/Topic | Item No | Checklist item | Reported on page No |
| --- | --- | --- | --- |
| Title and abstract | | | |
|  | 1a | Identification as a randomised trial in the title | Yes. Title page |
|  | 1b | Structured summary of trial design, methods, results, and conclusions (for specific guidance see CONSORT for abstracts) | Yes. Abstract |
| Introduction | | | |
| Background and objectives | 2a | Scientific background and explanation of rationale | Introduct. p4-5 |
|  | 2b | Specific objectives or hypotheses | Introduct. last section p5 |
| Methods | | | |
| Trial design | 3a | Description of trial design (such as parallel, factorial) including allocation ratio | Metods p5 |
|  | 3b | Important changes to methods after trial commencement (such as eligibility criteria), with reasons | NA |
| Participants | 4a | Eligibility criteria for participants | Methods last section p5 |
|  | 4b | Settings and locations where the data were collected | Setting and procedure p6, Assessment p8 |
| Interventions | 5 | The interventions for each group with sufficient details to allow replication, including how and when they were actually administered | Interventions p6- 7, figure 1 |
| Outcomes | 6a | Completely defined pre-specified primary and secondary outcome measures, including how and when they were assessed | Assessment p8, table I |
|  | 6b | Any changes to trial outcomes after the trial commenced, with reasons | No |
| Sample size | 7a | How sample size was determined | Power cal. p9-10 |
|  | 7b | When applicable, explanation of any interim analyses and stopping guidelines | None |
| Randomisation: |  |  | Procedure p6 |
| Sequence generation | 8a | Method used to generate the random allocation sequence |  |
|  | 8b | Type of randomisation; details of any restriction (such as blocking and block size) | Procedure p6 |
| Allocation concealment mechanism | 9 | Mechanism used to implement the random allocation sequence (such as sequentially numbered containers), describing any steps taken to conceal the sequence until interventions were assigned | Protection last section p8 |
| Implementation | 10 | Who generated the random allocation sequence, who enrolled participants, and who assigned participants to interventions | Procedure p6, protection p8 |
| Blinding | 11a | If done, who was blinded after assignment to interventions (for example, participants, care providers, those assessing outcomes) and how | Assessor blinding ‘Research design’ p5, Protection p8 |
|  | 11b | If relevant, description of the similarity of interventions | Health counselling p8 |
| Statistical methods | 12a | Statistical methods used to compare groups for primary and secondary outcomes | Statistics p9 |
|  | 12b | Methods for additional analyses, such as subgroup analyses and adjusted analyses | Comparison of effect sizes (cohens *d*) ‘Results’ p18, Explorative analysis of VO_2_-peak ‘Results’ p18 |
| Results | | | |
| Participant flow (a diagram is strongly recommended) | 13a | For each group, the numbers of participants who were randomly assigned, received intended treatment, and were analysed for the primary outcome | Flowchart Figure 2 |
|  | 13b | For each group, losses and exclusions after randomisation, together with reasons | Flowchart Figure 2 |
| Recruitment | 14a | Dates defining the periods of recruitment and follow-up | Figure1, table I, Assessment p8 |
|  | 14b | Why the trial ended or was stopped | Power cal p9-10 |
| Baseline data | 15 | A table showing baseline demographic and clinical characteristics for each group |  |
| Numbers analysed | 16 | For each group, number of participants (denominator) included in each analysis and whether the analysis was by original assigned groups | Figure 3, table 3, 4, 5 |
| Outcomes and estimation | 17a | For each primary and secondary outcome, results for each group, and the estimated effect size and its precision (such as 95% confidence interval) | Figure 6 |
|  | 17b | For binary outcomes, presentation of both absolute and relative effect sizes is recommended | - |
| Ancillary analyses | 18 | Results of any other analyses performed, including subgroup analyses and adjusted analyses, distinguishing pre-specified from exploratory | Explorative analysis of VO_2_-peak ‘Results’ p18 |
| Harms | 19 | All important harms or unintended effects in each group (for specific guidance see CONSORT for harms) | Results second section p10 |
| Discussion | | | |
| Limitations | 20 | Trial limitations, addressing sources of potential bias, imprecision, and, if relevant, multiplicity of analyses | Discussion last section p22 |
| Generalisability | 21 | Generalisability (external validity, applicability) of the trial findings | Discussion last section p22 |
| Interpretation | 22 | Interpretation consistent with results, balancing benefits and harms, and considering other relevant evidence | Discussion p19-20, Conclusions p22 |
| Other information | | | ISRCTN.org |
| Registration | 23 | Registration number and name of trial registry | [*http://www.isrctn.com/ISRCTN13816000*](http://www.isrctn.com/ISRCTN13816000) |
| Protocol | 24 | Where the full trial protocol can be accessed, if available | The initial protocol: BMJ Open <https://www.ncbi.nlm.nih.gov/pubmed/24189081>  Feasibility study:  <https://www.ncbi.nlm.nih.gov/pubmed/27900123> |
| Funding | 25 | Sources of funding and other support (such as supply of drugs), role of funders | Declarations – funding p24 |

*We strongly recommend reading this statement in conjunction with the CONSORT 2010 Explanation and Elaboration for important clarifications on all the items. If relevant, we also recommend reading CONSORT extensions for cluster randomised trials, non-inferiority and equivalence trials, non-pharmacological treatments, herbal interventions, and pragmatic trials. Additional extensions are forthcoming: for those and for up to date references relevant to this checklist, see [www.consort-statement.org](http://www.consort-statement.org/).
